# Combining performance and outcome indicators can be used in a standardized way: a pilot study of two multidisciplinary, full-scale major aircraft exercises

**DOI:** 10.1186/1757-7241-20-58

**Published:** 2012-08-28

**Authors:** Monica Rådestad, Heléne Nilsson, Maaret Castrén, Leif Svensson, Anders Rüter, Dan Gryth

**Affiliations:** 1 Karolinska Institutet, Department of Clinical Science and Education and Section of Emergency Medicine, Södersjukhuset, Stockholm, Sweden; 2 Centre for Teaching and Research in Disaster Medicine and Traumatology, Linköping University, Linköping, Sweden; 3 Karolinska Institutet, Department of Clinical Science and Education, Södersjukhuset, Stockholm, Sweden; 4 Sophiahemmet University College, Stockholm, Sweden; 5 Karolinska Institutet, Department of Physiology and Pharmacology and Section of Anaesthesiology and Intensive care, Stockholm, Sweden

**Keywords:** Airplane crash, Disaster preparedness, Disaster management, Disaster response, Educational model, Field exercise, Major incident, Simulation

## Abstract

**Background:**

Disaster medicine is a fairly young scientific discipline and there is a need for the development of new methods for evaluation and research. This includes full-scale disaster exercisers. A standardized concept on how to evaluate these exercises, could lead to easier identification of pitfalls caused by system-errors in the organization. The aim of this study was to demonstrate the feasibility of using a combination of performance and outcome indicators so that results can be compared in standardized full-scale exercises.

**Methods:**

Two multidisciplinary, full-scale exercises were studied in 2008 and 2010. The panorama had the same setup. Sets of performance indicators combined with indicators for unfavorable patient outcome were recorded in predesigned templates. Evaluators, all trained in a standardized way at a national disaster medicine centre, scored the results on predetermined locations; at the scene, at hospital and at the regional command and control.

**Results:**

All data regarding the performance indicators of the participants during the exercises were obtained as well as all data regarding indicators for patient outcome. Both exercises could therefore be compared regarding performance (processes) as well as outcome indicators. The data from the performance indicators during the exercises showed higher scores for the prehospital command in the second exercise 15 points and 3 points respectively. Results from the outcome indicators, patient survival and patient complications, demonstrated a higher number of preventable deaths and a lower number of preventable complications in the exercise 2010. In the exercise 2008 the number of preventable deaths was lower and the number of preventable complications was higher.

**Conclusions:**

Standardized multidisciplinary, full-scale exercises in different settings can be conducted and evaluated with performance indicators combined with outcome indicators enabling results from exercises to be compared. If exercises are performed in a standardized way, results may serve as a basis for lessons learned. Future use of the same concept using the combination of performance indicators and patient outcome indicators may demonstrate new and important evidence that could lead to new and better knowledge that also may be applied during real incidents.

## Background

The overall aim for medical responses to disasters and major incidents (MIs) is to achieve the best possible outcome for the most number of victims. However, in order to evaluate the outcome of a response you also need to assess the processes involved. To be able to do this there is a need for standardization and for sets of goals (bench-marks) reflecting what is to be considered as good and less good performance. These processes and goals must be developed in a way that allows them to be systematically studied, analyzed and with the possibility to compare results and experiences [1-3]. Several researchers have indicated the need for validated assessment methods performed in a scientifically genuine manner in order to measure the effectiveness of disaster medicine response [4-8].

In most healthcare systems the use of quality indicators are mandatory for determining standards and measuring quality. The challenge is to address indicators even in the field of disaster medicine. The use of measurable quality indicators to determine the level of performance against predetermined goals and objectives has been addressed in previous studies [9-11]. In these studies the quality indicators represent standards (times and content) of what is desirable performance of the management during MIs and disasters and were results from process- and concept modelling and/or opinion of an expert panel [10].

A few educational models for simulation of disaster management and MIs response are accepted and used in Sweden [12,13]. However, there are a limited number of full-scale exercises (FSE) and systematic and validated evaluation tools are still in the process of being developed. Therefore the competence and effectiveness of the emergency medical services (EMS) and hospital performance seldom are assessed in a way that allows results to be compared. The question arises whether a standardized evaluation methodology would be applicable in a FSE to assess if and how the management is related to the patient outcome. The aim of this study was to demonstrate the feasibility of using a combination of performance and outcome indicators so that results can be compared in standardized full-scale exercises.

## Methods

### Description of the exercises

A quantitative evaluation method was applied in two multidisciplinary, FSE (major aircraft accident) in two regions in Sweden. The two exercises studied were conducted in October 2008 and in April 2010 at two different airports. The time interval, 18 months, between the exercises is due to the willingness of the two different airports and county councils to participate in this study. According to national regulations, each international airport is mandated to perform an exercise every other year, and there were, to our knowledge, no other airport exercises during this time interval [14].

### Exercise scenario

The scenario was: an aircraft carrying passengers and crew that crashed during a landing attempt resulting in 99 respectively 100 victims (99 for the reason that one person, aimed to be figurant, felt sick on a late notice). In both exercises the participants, EMS and hospital personnel, were all familiar with the management structure (doctrine) at MI and disaster and expected to respond according to plans and procedures as stated in the national regulations issued by the Swedish National Board of Health and Welfare [15].

A total of 131 and 69 health care workers, respectively participated in the exercises. The exercises were conducted in real-time. All the participants were alerted and dispatched according to the disaster plan. The available resources in both exercise settings, ambulance, helicopter, health care, fire and rescue service, police and other responding agencies, were all defined in beforehand. Figurants acted as mock victims and had injuries that were appropriate to what was to be expected in this type of accident. Each victim had their injuries visualized on a figurant- card and all injuries had predetermined medical needs according to a specific template in the Emergo Train System® (ETS) victim bank [12].

**Figure 1:**
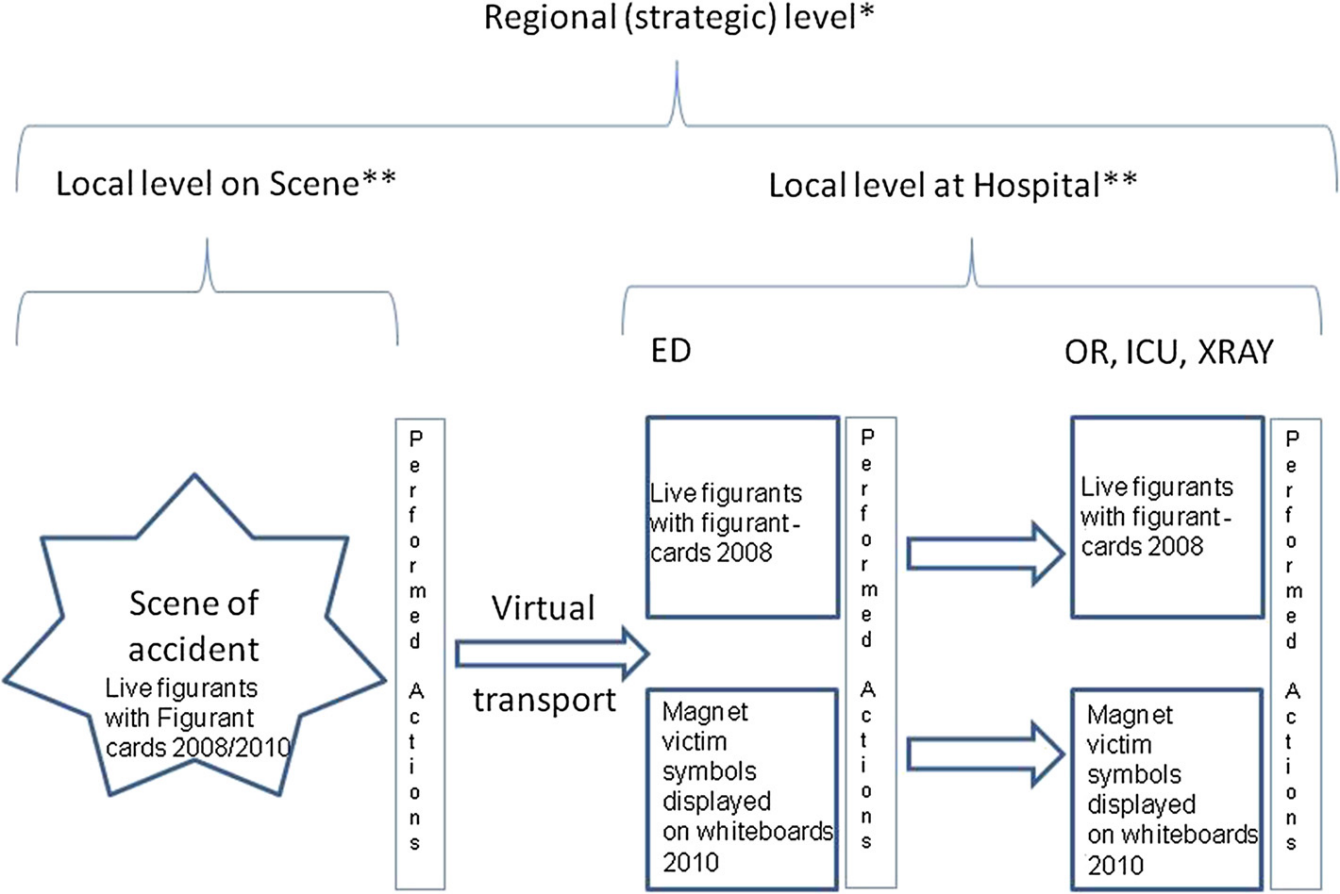
**Flow chart of victim distribution.** Overall design of two full-scale disaster exercises including the possibility to use figurant-cards, combined with tabletop simulation. *At the regional level results from performance indicators were obtained. **At the local level on scene and in hospital results from both performance indicators and patient outcome were obtained.

The victim’s conditions were expressed in physiological parameters. The basis for the victim bank has been developed on a national consensus among traumatologists. ETS is a simulation tool for education and training in disaster medicine which can be used both for table top exercises and field exercises as well as templates for evaluating results of performance and the treatment of the victims. A similar system is the Mass Casualty Simulation system (MACSIM) but with further developed injury card [13].

### Exercise design and mock victim distribution

**Figure 2:**
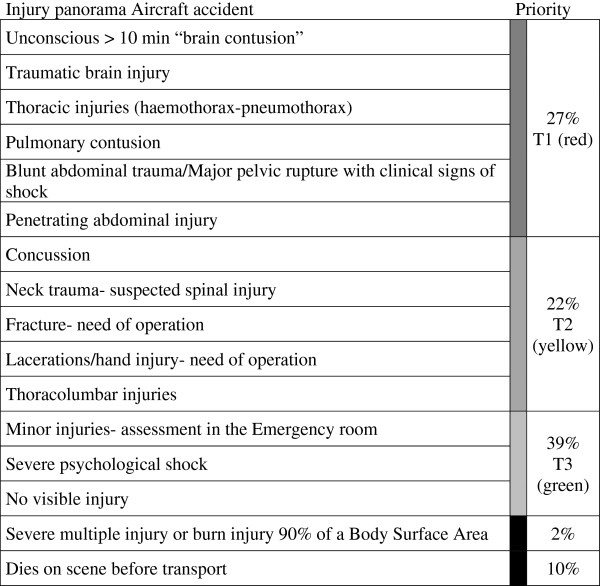
**Distribution of predetermined priorities of victims (n = 100) according to the injury panorama in ETS.** Triage categories were based on the physiological parameters obtained from the figurant-card as respiratory rate, pulse rate, systolic blood pressure and Glasgow Coma Scale. T1 (red) immediate, severely injured; T2 (yellow) urgent, moderately injured; T3 (green) not urgent, minor injured; (Black) dead.

Figure 1 shows how the exercise design was built up using the flexibility of a standardized victim set. The flow chart of victims is based on distribution from the scene of the accident through virtual transport to the hospital emergency department (ED). All victims were on-site triaged and treated by medical personnel and distributed to hospitals by the designated duty officer (DDO) at the strategic level of management (Figure 2). In 2008 live figurants were used to act as victims at the ED and in 2010 magnet victim symbols were used and displayed on whiteboards according to the simulation system [12].

Instead of actually transporting the victims to the hospital by ambulances, ambulances were in both settings used only at the scene of the accident, and for a short transportation to the point where victims data were reported to the receiving hospitals. This data, regarding measures performed on each victim, was at this point collected by trained nurses and reported by telephone to the hospital ED. Also, the expected time of arrival (according to real transportation time) was reported. This meant, that a mock-victim in 2008 and magnet victim symbol in 2010, were presented with exactly the same data as the mock-victim that had participated on-scene, could be presented at the ED. By using this method, fewer ambulances were needed at the scene of the accident although the realism was the same. In a real MI probably 25–30 ambulances would rapidly respond to the requirement through the dispatch centre. For obvious reason access to ambulances is limited in FSE. However, for testing the concept merely two hospitals participated, one in each exercise, and received 17 victims, respectively. Both hospitals were emergency hospitals, and had a capacity of 302 and 309 beds, respectively. Available intensive care unit beds were 13 and 12 respectively, and operating rooms were nine and ten respectively. The time period in hospital, for each victim, included the time spent at the ED, x-ray, surgery (OR) and the estimated time for post operative- and intensive care. Performance- and patient outcome indicators were included in the evaluation of both exercises.

### Data collected

The external evaluators were registered nurses and doctors certified as instructors in a Swedish national concept in disaster preparedness [15]. One or two evaluators were positioned at designated areas and used a protocol with sets of performance indicators as templates, all trained in a standardized way at a national disaster medicine centre (Table 1). These indicators were the same as those that had been used in previous studies [9-11].

**Table 1:** Sets of performance indicators and standards used for evaluation in full-scale disaster exercises

**Prehospital command and control**	**Standard (time frame in min)**
Putting on tabard*	Directly*
First report to dispatch	2
Content on first report	METHANE**
Formulate guidelines for response	3
Establish contract with strategic level of command	5
Liaison with fire and police	5
Second report from scene	10
Content of second report	Verifying first report. Indicating first patient transport
Establish level of medical ambition	10
First patient evacuated	15
Information to media on scene	30
**Regional command and control**	Standard (time frame in min)
Declaring major incident	1
Deciding level of preparedness	3
Decision on additional resources to scene	3
Deciding on receiving hospitals	5
Establishing contact with incident officers at scene	10
Deciding on guidelines for referring hospitals	10
Brief information to media	15
Formulate general guidelines in accordance with guidelines from scene	15
Make sure there is information for definitive referral guidelines	20
Evaluated if capacity of own organisation is sufficient	30
Notify guidelines on referring hospitals	40
**Hospital command and control**	Standard (time frame in min)
Decide on level of preparedness	3
Formulate guidelines for hospital response	15
Inform media	15
Give information about resources to strategic level	25
Ensuring that there is a medical officer in emergency operation	30
Estimate need of ICU beds	45
First information to staff	60
Estimate endurance of staff	90
Evaluate and report estimated shortage of own capacity	120
Evaluate influence on the daily hospital activities	120
Information plan for patients with postponed appointments and operations	180
**Regional and Hospital staff procedure skills**	Standard (time frame in min)
Assigning functions to all the staff members directly upon arrival	Directly on arrival
Placement in room according to function in staff	Directly
Designated telephone numbers	Directly
Introduction of newly arrived staff member	Maximum 1
Utilization of available equipment^a^	
Maximum 8 min for “staff briefing”	8
Content of “staff briefing”^b^	
Telephone discipline during “staff briefing”	Yes/No
Drawing and content of “staff schedule”	Yes/No
Summary after session, orally	Yes/No
Summary after session, written	Yes/No

* Vest, clearly labelled for identification of medical and ambulance staff.

** **M**ajor incident declared, **E**xact location, **T**ype of incident, **H**azards, **A**ccessibility, **N**umber of casualties, **E**mergency Services required. Acronym for the content, defined in the Major Incident Medical Management and Support Course (MIMMS).

^a^ Equipment available: whiteboard, flipchart, fax, and computer.

^b^Reports from all functions, summarizing, assigning new tasks, time for next briefing.

The protocols were used for evaluation of performance at two different levels of medical management, regional level (in international literature often called strategic or gold level) and local level i.e. on-scene and in hospital (Figure 1).

The *performance indicators* covered early decision- making (management skills), as well as staff performance. The level was scored by giving 0, 1, or 2 points where 0 point was given if the performance was not timely or adequate, 1 point if the result was somewhat correct and 2 points for a timely and by content, correct performance. Each template contained 11 indicators, thereby allowing for 22 points being maximum achievable level of result (Table 1). Eleven out of 22 points where considered satisfactory.

The *outcome indicator*, patient outcome, was evaluated in terms of risk for preventable death or complication [12]. Each victim was assigned to specific measures in order to be expected to have a favorable outcome, all according to templates that are included in the ETS. If these treatments were not performed or performed too late, according to the ETS stipulated timeframe, the patient will have a preventable complication or death. In ETS every specific treatment or transfer are performed according to a “real-time” approach.

All logistical and clinical data were registered at the scene of the accident and in hospital, regarding the care performed in real time, by participants who had been trained and instructed in ETS template.

## Results

All data regarding the performance indicators of the participants during the exercises were obtained as well as all data regarding indicators for patient outcome. Both exercises could therefore be compared regarding performance (process) as well as outcome (result) indicators.

### Performance indicators

All result were on approved level except for prehospital command in the first exercise 2008 that scored 3 points compared to the second exercise 2010 that scored 15 points. Apart from this result, all other results were on the same level in the two exercises (Table 2). In common for the regional management groups is that there were low scores for establishing contact with incident officers at the scene, first information to media and estimating if resources within their own organization were adequate for managing the accident. All management groups (hospital and strategic) received high score in performance of staff skills as well as regarding correct and timely decisions.

**Table 2:** Results based on templates of performance indicators, expressed in points

** Management level**	**Exercise 2008**		**Exercise 2010**	
	**Category**		**Category**	
	Command & control*	Staff procedure* skills	Command & Control*	Staff procedure* skills
Prehospital (Local level)	3	Not assessed	15	Not assessed
Regional	15	17	18	21
Hospital (Local level)	17	21	17	20

*Maximum score was 22 points in each category where 11 different indicators were given 0, 1 or 2 points. Score: Correct decision and in right time. Correct = 2 points, Partly correct = 1 points, Incorrect = 0 points.

### Outcome indicators

Results from the patient outcome indicators, that is preventable death and/or preventable complications, demonstrated a higher number of preventable deaths in exercise 2010, (n = 7) compared in the 2008 exercise (n = 5). Regarding the preventable complications the results were opposite with a higher number in the 2008 exercise (n = 9) compared to the 2010 exercise (n = 5) (Table 3). Out of 99 respectively 100 victims, the participating hospital in each exercise received totally 17 injured victims prioritized as T1 and T2. The numbers of ambulances were according to plan and were considered to be on an adequate level (simulated as previously described). The first victim arrived at the ED 100 min respectively 105 min after the accident, for the last victim this was 235 min and 273 min respectively.

**Table 3:** Patient outcome expressed as preventable complications and preventable death in two, full-scale disaster exercises

	**Exercise 2008**	**Exercise 2010**
Preventable Complication	53% (9/17*)	29% (5/17*)
Preventable Death	29% (5/17*)	41% (7/17*)

*All 17 victims receiving the participating hospital were at risk, according to ETS template, for having unfavorable outcome expressed as preventable complication or preventable death.

## Discussion

This study demonstrates the possibility of conducting standardized exercises with built-in evaluation methodology that can produce comparable results. The discussion in this paper focuses more on the methodology than the results from the exercises per se. A prerequisite for results from evaluations to be comparable is that the exercises are evaluated the same way, making it possible to obtain valid data to be recorded. In the present study we have demonstrated the possibility to apply both performance and outcome indicators in multidisciplinary, FSE. Indicators should be based on well-developed standards established and accepted by the organization to ensure comparability and reproducibility. The selected indicators were derived from a national concept and process modeling conducted by the National Board of Health and Welfare in Sweden [15]. Idvall et al. describes this as ‘the key to good quality indicators’ and the only certain way of knowing what is good or less good by comparing performance against the standard [16]. Furthermore, Idvall et al. clarifies that the most important indicator is the patient outcome, and it is a product influenced by all activities in an organization and should be measured together with other indicators. Standards (bench-marks) for evaluation methods that examine and describe the relation between the performance in disaster management and the patient outcome are yet to be described [17].

The evaluation model exposed several challenges faced in the initial decision-making procedures that were repeatedly observed and had major impact on patient outcome. For example, insufficient reporting and inconsistent coordination among responders that limited the ability to evacuate severely injured victims and resulted in comparatively high numbers of preventable death and complications. Despite good access to available ambulances in both exercises failure of rapid evacuation indicates that decisions made in this context were less than optimal which could possibly have been reflected in the patient outcome. By using the proposed model each agency will have increased possibilities to specifically identify what part of the command and control that needs to be improved. The fact that the difference between the prehospital command and control, as illustrated in Table 2, did not show in patient outcome can however, not be explained and this is something that may have to be addressed using different research methods. At this stage, after only a few exercises, it is still too early to assess any relationship between performance and outcome. There is also a need to study if timely decisions or actions are more important than the content of the performance. In the present study the two prehospital organizations had different training concepts with regard to command and control, even though they had to follow the same regulations in performing [15].

Efficient disaster management requires information sharing, follow up and coordinated decision-making among involved agencies. Jufferman and Bierens studied five national disaster responses and noted that many shortages are repeated despite changes in protocols, legislation and organizations [6]. For this reason there is a need for interagency training to improve operational decisions. Circumstances at the scene such as time -period, geography, weather conditions, reliable communication systems and security are other essential factors that may affect the response and therefore have to be considered in the assessment. This problem was recently described after a real aircraft incident where there were difficulties to evacuate the victims and this delayed the distribution of victims to hospitals [18].

The use of standardized education and training models, with built-in evaluation possibilities, allows comparisons and could lead to better knowledge and improve disaster medicine evidence in management system. In this study the first exercise was reproduced and the same type of evaluation, obtaining the same types of comparable results, was used in the second exercise. To be able to draw valid conclusions, the number of exercises performed must be higher. But this is also depending on number of indicators and methodology chosen. However, in our opinion, we must already start now to collect data as described in this study in order for the lessons learned in the processes not to be delayed further. The rational for this is the development of a robust design of the evaluation model used in the present study.

In the planning phase consideration was, in this study, taken both to each hospitals request of participation as well as their concerns regarding financing of staff which had impact upon the exercise design (Figure 1). In our view, the difference in methodology regarding live figurants vs. virtual victims used at hospital, shows the models robustness in several settings. It is our belief that before engaging several hospitals in this type of structured exercises it should be prioritized to evaluate the first steps of the process. When this model becomes more accepted, additional units that can be trained and evaluated should be introduced.

### Significance of this study for the future

To achieve greater acceptance methods with valid indicators need to be promoted by the health care providers. By using the same indicators in the evaluation of real incidents, in relation to available patient data, it would be possible to not only compare the performance indicators but also to validate the outcome indicators chosen in the exercises. The comparison with real incidents will give clues if we are educating and training the staff in the best way, and comparing results will provide information about possible improvements of the pedagogic educational simulation system used today. Knowledge that will émerge from this will no doubt, improve the possibility to conduct measurements in order to improve the disaster response before a real incident occurs. It will also reduce the time consumption for planning and after-action report writing.

Indicators are now gradually introduced in different regions in Sweden, so far, for evaluating the effect of training in order to measure quality in performance related to the management doctrine, reflected by regional regulations, supported by the Swedish National Board of Health and Welfare. Also in an international study results from using the same sets of indicators has recently been published [19].

In this study we have not considered structure indicators relating to staff, time and financial costs. Results from future studies with a systematic approach to the structure indicators will provide important information on how train and exercises should be conducted in the best way.

### Limitations

Competency and quality between different levels of management and organisations in this paper can depend on variation of personnel’s professional qualifications, education, experience and training in disaster medicine and might be a limitation. This applies also to the evaluators. Different evaluators may give different scoring for different processes. There is also a need to study if timely decisions or actions are more important than the content of the performance.The used simulation system may have limitations for evaluating patient outcome, but until studies from real incidents (where performance indicators as well as outcome indicators) are available we believe that this method will provide valid results.

The basis to use 11 points as acceptable level for measuring performance is based on results from training and examination sessions in command and control during several years of experience [9-11]. However, it could well be that any of the indicators could be more important than the others. Correlations studies may give answer to this. It may also well be that the indicators in the future must be weighted.

The figurant in the first exercise, that fell sick on late notice did not simulate a critical injured patient and was not intended to be sent to the participating hospital. Therefore we do not believe that this has any effect on the results.

Cost effectiveness, in regards to this subject, was not addressed in this study. This is due to the lack of systematic documentation of total costs for all involved staff including time for planning as well as the actual exercise.

## Conclusions

Standardized FSE in different settings can be conducted and evaluated with performance indicators combined with outcome indicators enabling results from exercises to be compared. If exercises are performed in a standardized way, results may serve as a basis for lessons learned. Future use of the same concept using the combination of performance indicators and patient outcome indicators may demonstrate new and important evidence that could lead to new and better knowledge that also may be applied during real incidents.

## Competing interests

This work was performed by Stockholm Prehospital Centre (SPC) in cooperation with the Centre of Teaching in Disaster Medicine and Traumatology (KMC), Linköping University, Sweden and the Sophiahemmet University College, Stockholm, Sweden. The authors declare that they have no competing interests.

## Authors’ contributions

MR was involved in the study design, exercise data collection, analysis, and manuscript writing. HN was involved in the study design and data collection. MC and LS contributed to the finalization of the manuscript. AR and DG was involved in the study design and took an active part in the data collection, analysis and manuscript writing, revision and editing. All authors read and approved the final version of the manuscript.
